# Aggregation operators of neutrosophic linguistic numbers for multiple attribute group decision making

**DOI:** 10.1186/s40064-016-3247-5

**Published:** 2016-10-01

**Authors:** Jun Ye

**Affiliations:** Department of Electrical and Information Engineering, Shaoxing University, 508 Huancheng West Road, Shaoxing, 312000 Zhejiang Province China

**Keywords:** Neutrosophic linguistic number, Neutrosophic linguistic number weighted arithmetic average (NLNWAA) operator, Neutrosophic linguistic number weighted geometric average (NLNWGA) operator, Group decision making

## Abstract

Based on the concept of neutrosophic linguistic numbers (NLNs) in symbolic neutrosophic theory presented by Smarandache in 2015, the paper firstly proposes basic operational laws of NLNs and the expected value of a NLN to rank NLNs. Then, we propose the NLN weighted arithmetic average (NLNWAA) and NLN weighted geometric average (NLNWGA) operators and discuss their properties. Further, we establish a multiple attribute group decision-making (MAGDM) method by using the NLNWAA and NLNWGA operators under NLN environment. Finally, an illustrative example on a decision-making problem of manufacturing alternatives in the flexible manufacturing system is given to show the application of the proposed MAGDM method.

## Background

In decision theory, decision-making method is one of important research topics. Then various decision-making methods has been proposed and applied widely to engineering, economics, and management fields. However, in complex decision-making problems, decision makers (DMs) may give the qualitative evaluation for attributes by linguistic variables (LVs) due to the uncertainty of decision environment and difference of DMs’ cultural and knowledge background. Hence, Zadeh (1975) firstly presented the LV concept and its application in fuzzy reasoning. Later, linguistic decision analyses were introduced to deal with decision-making problems with linguistic information (Herrera et al. 1996; Herrera and Herrera-Viedma 2000). Then, a linguistic hybrid arithmetic average operator was used for multiple attribute group decision-making (MAGDM) problems with linguistic information (Xu 2006a). Further, goal programming models were put forward to handle multiple attribute decision-making (MADM) problems under linguistic environment (Xu 2006b). Also, the uncertain linguistic ordered weighted averaging (ULOWA) and uncertain linguistic hybrid aggregation (ULHA) operators were presented for dealing with MAGDM problems with uncertain linguistic information (Xu 2004). Some induced uncertain linguistic ordered weighted average (IULOWA) operators were further developed for MAGDM problems with uncertain linguistic information (Xu 2006c).

However, incomplete, indeterminate, and inconsistent information often exists in real life. To express this kind of information, Smarandache (1998, 2013, 2014) proposed the concept of a neutrosophic number (NN), denoted by *B* = *t* + *vI*, which consists of its determinate part *t* and its indeterminate part *vI*. Then, neutrosophic sets and NNs (Smarandache 1998, 2013, 2014) are two different branches in neutrosophic theory. Here, NN can express incomplete, indeterminate and inconsistent information by the determinate and indeterminate parts, which exists commonly in real life, while existing linguistic information cannot express indeterminate and inconsistent information. Since NN can effectively express incomplete and indeterminate information, Ye (2015) developed a bidirectional projection method for MAGDM problems with NNs. Ye (2016) proposed a MAGDM method with NNs, including a de-neutrosophication process and a possibility degree ranking method for NNs. Kong et al. (2015) presented a cosine similarity measure between NNs and applied it to the misfire fault diagnosis of gasoline engines.

Because of the ambiguity of people’s thinking about the complex objective things in the real world, linguistic evaluation in complex decision-making problems may easily express and better deal with the incomplete and indeterminate information than numerical evaluation. However, existing linguistic variables cannot express indeterminate and inconsistent information, hence one needs to introduce a expression form of indeterminate linguistic information to overcome the difficulty of existing linguistic expression for indeterminate information. Recently, Smarandache introduced the concept of a neutrosophic linguistic number (NLN) expressed by *l*_*t*+*vI*_ in symbolic neutrosophic theory (Smarandache 2015), where *t* + *vI* is NN. Unfortunately, there are not operational laws of NLNs and their application till now. To study these problems, the objects of this paper are: (1) to define basic operational laws of NLNs and the expected value of a NLN for ranking NLNs, (2) to propose the NLN weighted arithmetic average (NLNWAA) and NLN weighted geometric average (NLNWGA) operators and to discuss their properties, and (3) to establish a MAGDM method based on the NLNWAA and NLNWGA operators under NLN environment. The main advantages of the proposed method are able to express and handle indeterminate linguistic information in linguistic decision-making environments and extend the existing NN decision-making methods (Ye 2015, 2016) to NLN decision-making method.

The remainder of this paper is organized as follows. Section “Preliminaries of LSs, NNs and NLNs” briefly reviews the basic concepts of LVs, linguistic sets (LSs), NNs, and NLNs. The operational laws of NLNs and the expected value of a NLN are defined in “Operational laws and expected value of NLNs” section. “Weighted aggregation operators for NLNs” section develops NLNWAA and NLNWGA operators of NLNs and discusses their properties. In “MAGDM method using the NLNWAA and NLNWGA operators” section, a MAGDM method based on the NLNWAA and NLNWGA operators is developed under NLN environment. In “Illustrative example” section shows the application of the presented method. Conclusions and future research are contained in “Conclusion” section.

## Preliminaries of LSs, NNs and NLNs

### The concepts of LV and LS

Zadeh (1975) firstly proposed the concept of LV in 1975.

Let *L* = {*L*_0_, *L*_1_, …, *L*_*s*−1_} be a finitely linguistic term set with cardinality *s*, where *L*_*i*_ in the linguistic term set *L* is a LV and *s* is an odd value. For instance, taking *s* = 7, one can specify a LS *L* = {*L*_0_, *L*_1_, *L*_2_, *L*_3_, *L*_4_, *L*_5_, *L*_6_} = {extremely poor, very poor, poor, medium, good, very good, extremely good}.

Then, any two LVs *L*_*i*_ and *L*_*j*_ in a LS *L* should satisfy the following properties (Herrera et al. 1996; Herrera and Herrera-Viedma 2000):

1. Ordering: *L*_*i*_ ≥ *L*_*j*_ if *i* ≥ *j*;
2. Negation operator: Neg(*L*_*i*_) = *L*_*s*−1−*i*_;
3. Maximum operator: Max(*L*_*i*_, *L*_*j*_) = *L*_*i*_ if *i* > *j*;
4. Minimum operator: Min(*L*_*i*_, *L*_*j*_) = *L*_*j*_ if *i* > *j*.

To minimize the linguistic information loss in the operational process, the discrete LS *L* = {*L*_0_, *L*_1_, *L*_2_, *L*_3_, *L*_4_, *L*_5_, *L*_6_} can be generalized to a continuous LS $\bar{L} = \{ L_{\theta } |\theta \in R\}$, which satisfied the above-mentioned characteristics.

For any two LVs *L*_*i*_ and *L*_*j*_ for $L_{i} ,L_{j} \in \bar{L}$, Xu (2006a, b) defined the following operational laws:

1. $$ \rho L_{i} = L_{\rho \times i} ,\quad \rho \ge 0; $$
2. $$ L_{i} + L_{j} = L_{i + j} ; $$
3. $$ L_{i} \times L_{j} = L_{i \times j} ; $$
4. $$ L_{i} /L_{j} = L_{i/j} ; $$
5. $$ \left( {L_{i} } \right)^{\rho } = L_{{i^{\rho } }} ,\quad \rho \ge 0. $$

### Some concepts of NNs and NLNs

NN proposed by Smarandache (1998, 2013, 2014) consists of the determinate part *t* and the indeterminate part *vI*, which is denoted by *B* = *t* + *vI*, where *t* and *v* are real numbers, and *I* is indeterminacy, such that *I* ^*n*^ = *I* for *n* > 0, 0 × *I* = 0, and *bI*/*nI* = undefinition for any real number *n*.

For example, assume that there is a NN *B* = 3 + 2*I*. If *I* ∈ [0, 0.3], it is equivalent to *B* ∈ [3, 3.6] for sure *B* ≥ 3, this means that its determinate part is 3 and its indeterminate part is 2*I* for the indeterminacy *I* ∈ [0, 0.3] and the possibility for the number “*B*” is within the interval [3, 3.6].

Let *B*_1_ = *t*_1_ + *v*_1_ *I* and *B*_2_ = *t*_2_ + *v*_2_ *I* be two NNs for *t*_1_, *v*_1_, *t*_2_, *v*_2_ ∈ *R* (all real numbers). The operational relationship for *B*_1_ and *B*_2_ is as follows (Smarandache 1998, 2013, 2014):

1. *B*_1_ + *B*_2_ = *t*_1_ + *t*_2_ + (*v*_1_ + *v*_2_)*I*;
2. *B*_1_ − *B*_2_ = *t*_1_ − *t*_2_ + (*v*_1_ − *v*_2_)*I*;
3. *B*_1_ × *B*_2_ = *t*_1_ *t*_2_ + (*t*_1_ *v*_2_ + *v*_1_ *t*_2_ + *v*_1_ *v*_2_)*I*;
4. *B*_1_^2^ = (*t*_1_ + *v*_1_ *I*)^2^ = *t*_1_^2^ + ((*t*_1_ + *v*_1_)^2^ − *t*_1_^2^)*I*;
5. $$ \frac{{B_{1} }}{{B_{2} }} = \frac{{t_{1} + v_{1} I}}{{t_{2} + v_{2} I}} = \frac{{t_{1} }}{{t_{2} }} + \frac{{t_{2} v_{1} - t_{1} v_{2} }}{{t_{2} (t_{2} + v_{2} )}} \cdot I $$ for *t*_2_ ≠ 0 and *t*_2_ ≠ −*v*_2_;
6. $$ \sqrt {B_{1} } = \sqrt {t_{1} + v_{1} I} = \left\{ \begin{aligned} \sqrt {t_{1} } - (\sqrt {t_{1} } + \sqrt {t_{1} + v_{1} } )I \hfill \\ \sqrt {t_{1} } - (\sqrt {t_{1} } - \sqrt {t_{1} + v_{1} } )I \hfill \\ - \sqrt {t_{1} } + (\sqrt {t_{1} } + \sqrt {t_{1} + v_{1} } )I \hfill \\ - \sqrt {t_{1} } + (\sqrt {t_{1} } - \sqrt {t_{1} + v_{1} } )I \hfill \\ \end{aligned} \right.. $$

Let *B* = *t* + *vI* be a NN. If *t*, *v* ≥ 0, then *B* is called the positive NN.

In the following, all NNs are considered as positive and are called NNs for short, unless they are stated.

In 2015, Smarandache introduced the concept of NLN expressed by *l*_*t*+*vI*_ in symbolic neutrosophic theory (Smarandache 2015), where *t* + *vI* is NN, *t* and *v* are real numbers, and *I* is indeterminacy.

## Operational laws and expected value of NLNs

In this section, we give the operational laws of NLNs and the expected value of a NLN for ranking NLNs.

### **Definition 1**

Assume that $\bar{l}_{1} = l_{{t_{1} + v_{1} I}}$ and $\bar{l}_{2} = l_{{t_{2} + v_{2} I}}$ are two NLNs, then the operational laws are defined as follows:

1. $$ \bar{l}_{1} + \bar{l}_{2} = l_{{t_{1} + t_{2} + (v_{1} + v_{2} )I}} ; $$
2. $$ \bar{l}_{1} - \bar{l}_{2} = l_{{t_{1} - t_{2} + (v_{1} - v_{2} )I}} ; $$
3. $$ \bar{l}_{1} \times \bar{l}_{2} = l_{{t_{1} t_{2} + (t_{1} v_{2} + t_{2} v_{1} + v_{1} v_{2} )I}} ; $$
4. $$ \frac{{\bar{l}_{1} }}{{\bar{l}_{2} }} = {l}_{{\frac{{t_{1} }}{{t_{2} }} + \frac{{t_{2} v_{1} - t_{1} v_{2} }}{{t_{2} (t_{2} + v_{2} )}} \cdot I}} $$ for *t*_2_ ≠ 0 and *t*_2_ ≠ − *v*_2_;
5. $$ \rho \bar{l}_{1} = {l}_{{\rho t_{1} + \rho v_{1} I}} $$ for *ρ* ≥ 0;
6. $$ \bar{l}_{1}^{\rho } = l_{{t_{1}^{\rho } + [(t_{1} + v_{1} )^{\rho } - t_{1}^{\rho } ]I}} $$ for *ρ* ≥ 0.

Clearly, the above operational results are still NLNs.

Then, we define an expected value of a NLN, which is an important index to rank NLNs in the following decision-making problems.

### **Definition 2**

Let *L* = {*l*_0_, *l*_1_, …, *l*_*s*−1_} be a finitely linguistic term set with cardinality *s* and $\bar{l} = l_{t + vI}$ for *L* be a NLN and *I* ∈ [inf *I*, sup *I*]. Then, an expected value of the NLN $\bar{l}$ can be represented as

1 $$ E(\bar{l}) = \frac{(t + v\inf I) + (t + v\sup I)}{2(s - 1)}. $$

Obviously, the bigger the value of *E*($\bar{l}$) is, the greater the corresponding NLN $\bar{l}$ is.

Based on Definition 2, a ranking method for NLNs can be given below.

### **Definition 3**

Let $\bar{l}_{1}$ and $\bar{l}_{2}$ be two NLNs. Then, their ranking can be defined as follows:

1. If *E*($$ \bar{l}_{1} $$) > *E*($$ \bar{l}_{2} $$), then $$ \bar{l}_{1} $$ ≻ $$ \bar{l}_{2} $$;
2. If *E*($$ \bar{l}_{1} $$) = *E*($$ \bar{l}_{2} $$), then $$ \bar{l}_{1} $$ = $$ \bar{l}_{2} $$.

### *Example 1*

Let $\bar{l}_{1}$ = ${l}_{3 + 2I}$ and $\bar{l}_{2}$ = ${l}_{2 + 3I}$ be two NLNs for *I* ∈ [0.1, 0.3] and the cardinality of linguistic term sets *L* be *s* = 7. Then, in this case the ranking order between $\bar{l}_{1}$ and $\bar{l}_{2}$ is given as follows:

According to Eq. (1) we have *E*($\bar{l}_{1}$) = 0.5667 > *E*($\bar{l}_{2}$) = 0.4333, Hence, $\bar{l}_{1}$ ≻ $\bar{l}_{2}$.

## Weighted aggregation operators for NLNs

Weighted aggregation operator is an important tool for information aggregation, which can capture the expressed interrelationship of the individual arguments. Based on the operational laws in Definition 1, this section proposes the NLNWAA and NLNWGA operators to aggregate NLNs, which are usually utilized in decision-making problems.

### NLNWAA operator

#### **Definition 4**

Let $\bar{l}_{j}$ (*j* = 1, 2, …, *n*) be a collection of NLNs. The NLNWAA operator is defined by

2 $$ NLNWAA\left( {\bar{l}_{1} ,\bar{l}_{2} , \ldots ,\bar{l}_{n} } \right) = \sum\limits_{j = 1}^{n} {w_{j} \bar{l}_{j} } , $$

where *w*_*j*_ is the weight of $\bar{l}_{j}$ (*j* = 1, 2, …, *n*) with *w*_*j*_ ∈ [0, 1] and $\sum\nolimits_{j = 1}^{n} {w_{j} = 1}$.

#### **Theorem 1**

Let $\bar{l}_{j} \,\left( {j = 1,2, \ldots ,n} \right)$ *be a collection of NLNs. Then by Eq.* (2) *and the operational laws in Definition 1, we have the following aggregation formula:*

3 $$ NLNWAA\left( {\bar{l}_{1} ,\bar{l}_{2} , \ldots ,\bar{l}_{n} } \right) = {l}_{{\sum\nolimits_{j = 1}^{n} {w_{j} t_{j} + I\sum\nolimits_{j = 1}^{n} {w_{j} v_{j} } } }} , $$

*where w*_*j*_ *is the weight of* $\bar{l}_{j} \,\left( {j = 1,2, \ldots ,n} \right)$ *, satisfying* $w_{j} \in \left[ {0,1} \right]$ *and* $\sum\nolimits_{j = 1}^{n} {w_{j} = 1}$.

Obviously, the proof of Eq. (3) can be easily obtained according to the operational laws in Definition 1. Hence, its proof is omitted here.

Especially if $w_{j} = 1/n$ for $j = 1,2, \ldots ,n$, then the NLNWAA operator is reduced to a NLN arithmetic average operator.

Then, the NLNWAA operator shows the following properties:

1. Idempotency: Let $\bar{l}_{j} \,(j = 1,2, \ldots ,n)$ be a collection of NLNs. Then there is $NLNWAA\left( {\bar{l}_{1} ,\bar{l}_{2} , \ldots ,\bar{l}_{n} } \right) = \bar{l}$ if $\bar{l}_{j} \,(j = 1,2, \ldots ,n)$ is equal, i.e., $\bar{l}_{j} = \bar{l}$ for $j = 1,2, \ldots ,n.$
2. Monotonicity: Let $\bar{l}_{j} \,(j = 1,2, \ldots ,n)$ be a collection of NLNs. Then there is $NLNWAA\left( {\bar{l}_{1} ,\bar{l}_{2} , \cdots ,\bar{l}_{n} } \right) \le NLNWAA\left( {\bar{l}_{1}^{*} ,\bar{l}_{2}^{*} , \cdots ,\bar{l}_{n}^{*} } \right)$ if $\bar{l}_{j} \le \bar{l}_{j}^{*}$ for $j = 1,2, \ldots ,n.$
3. Boundedness: Let $\bar{l}_{j} \,(j = 1,2, \ldots ,n)$ be a collection of NLNs and $\bar{l}_{{\rm min} } = \hbox{min} (\bar{l}_{1} ,\bar{l}_{2} , \ldots ,\bar{l}_{n} )$ and $\bar{l}_{{\rm max} } = \hbox{max} (\bar{l}_{1} ,\bar{l}_{2} , \ldots ,\bar{l}_{n} )$ for $j = 1,2, \ldots ,n.$ Then there is $\bar{l}_{{\rm min} } \le NLNWAA\left( {\bar{l}_{1} ,\bar{l}_{2} , \ldots ,\bar{l}_{n} } \right) \le \bar{l}_{{\rm max} } .$

Since the above properties are obvious, their proofs are omitted here.

### NLNWGA operator

#### **Definition 5**

Let $\bar{l}_{j}$ (*j* = 1, 2, …, *n*) be a collection of NLNs. Then the NLNWGA operator is defined as

4 $$ NLNWGA\left( {\bar{l}_{1} ,\bar{l}_{2} , \ldots ,\bar{l}_{n} } \right) = \prod\limits_{j = 1}^{n} {\bar{l}_{j}^{{w_{j} }} } , $$

where *w*_*j*_ is the weight of $\bar{l}_{j}$ (*j* = 1, 2, …, *n*), satisfying *w*_*j*_ ∈ [0, 1] and $\sum\nolimits_{j = 1}^{n} {w_{j} = 1}$.

#### **Theorem 2**

*Let* $\bar{l}_{j} \,(j = 1,2, \ldots ,n)$ *be a collection of NLNs. by Eq.* (4) *and the operational laws in Definition 1, we have the following aggregation formula:*

5 $$ NLNWGA(\bar{l}_{1} ,\bar{l}_{2} , \ldots ,\bar{l}_{n} ) = {l}_{{\prod\nolimits_{j = 1}^{n} {t_{j}^{{w_{j} }} + \left( {\prod\nolimits_{j = 1}^{n} {(t_{j} + v_{j} )^{{w_{j} }} } - \prod\nolimits_{j = 1}^{n} {t_{j}^{{w_{j} }} } } \right)I} }} , $$

*where w*_*j*_ *is the weight of* $\bar{l}_{j} \,(j = 1,2, \ldots ,n)$ *, satisfying* $w_{j} \in \left[ {0,1} \right]$ *and* $\sum\nolimits_{j = 1}^{n} {w_{j} = 1} .$

#### *Proof*

The proof of Eq. (5) can be given by mathematical induction.

1. If *n* = 2, then
  6 $$ \begin{aligned} \bar{l}_{1}^{{w_{1} }} \times \bar{l}_{2}^{{w_{2} }} & = {l}_{{\left( {t_{1}^{{w_{1} }} + \left( {(t_{1} + v_{1} )^{{w_{1} }} - t_{1}^{{w_{1} }} } \right)I} \right) \times \left( {t_{2}^{{w_{2} }} + \left( {(t_{2} + v_{2} )^{{w_{2} }} - t_{2}^{{w_{2} }} } \right)I} \right)}} \\ & = {l}_{{t_{1}^{{w_{1} }} t_{2}^{{w_{2} }} + t_{1}^{{w_{1} }} \left( {(t_{2} + v_{2} )^{{w_{2} }} - t_{2}^{{w_{2} }} } \right)I + t_{2}^{{w_{2} }} \left( {(t_{1} + v_{1} )^{{w_{1} }} - t_{1}^{{w_{1} }} } \right)I + \left( {(t_{2} + v_{2} )^{{w_{2} }} - t_{2}^{{w_{2} }} } \right)\left( {(t_{1} + v_{1} )^{{w_{1} }} - t_{1}^{{w_{1} }} } \right)I}} \\ & = {l}_{{t_{1}^{{w_{1} }} t_{2}^{{w_{2} }} + \left( {t_{1}^{{w_{1} }} (t_{2} + v_{2} )^{{w_{2} }} - t_{1}^{{w_{1} }} t_{2}^{{w_{2} }} } \right)I + \left( {t_{2}^{{w_{2} }} (t_{1} + v_{1} )^{{w_{1} }} - t_{2}^{{w_{2} }} t_{1}^{{w_{1} }} } \right)I + \left( {(t_{2} + v_{2} )^{{w_{2} }} (t_{1} + v_{1} )^{{w_{1} }} - t_{2}^{{w_{2} }} (t_{1} + v_{1} )^{{w_{1} }} - t_{1}^{{w_{1} }} (t_{2} + v_{2} )^{{w_{2} }} + t_{1}^{{w_{1} }} t_{2}^{{w_{2} }} } \right)I}} \\ & = {l}_{{t_{1}^{{w_{1} }} t_{2}^{{w_{2} }} + \left( {(t_{2} + v_{2} )^{{w_{2} }} (t_{1} + v_{1} )^{{w_{1} }} - t_{1}^{{w_{1} }} t_{2}^{{w_{2} }} } \right)I}} . \\ \end{aligned} $$
2. If *n* = *k*, by using Eq. (5), we obtain
  7 $$ NLNWGA(\bar{l}_{1} ,\bar{l}_{2} , \ldots ,\bar{l}_{k} ) = {l}_{{\prod\nolimits_{j = 1}^{k} {t_{j}^{{w_{j} }} + \left( {\prod\nolimits_{j = 1}^{k} {(t_{j} + v_{j} )^{{w_{j} }} - \prod\nolimits_{j = 1}^{k} {t_{j}^{{w_{j} }} } } } \right)I} }} . $$
3. If *n* = *k* + 1, by using Eqs. (6) and (7), we obtain
  $$ \begin{aligned}& NLNWGA(\bar{l}_{1} ,\bar{l}_{2} , \ldots ,\bar{l}_{k + 1} ) \\& = {l}_{{\left( {\prod\nolimits_{j = 1}^{k} {t_{j}^{{w_{j} }} + \left( {\prod\nolimits_{j = 1}^{k} {(t_{j} + v_{j} )^{{w_{j} }} } - \prod\nolimits_{j = 1}^{k} {t_{j}^{{w_{j} }} I} } \right)} } \right)\left( {t_{k + 1}^{{w_{k + 1} }} + (t_{k + 1} + v_{k + 1} )^{{w_{k + 1} }} - t_{k + 1}^{{w_{k + 1} }} } \right)I}} \\ & = l_{{\prod\nolimits_{j = 1}^{k} {t_{j}^{{w_{j} }} t_{k + 1}^{{w_{j + 1} }} } + \prod\nolimits_{j = 1}^{k} {t_{j}^{{w_{j} }} \left( {(t_{k + 1} + v_{k + 1} )^{{w_{k + 1} }} - t_{k + 1}^{{w_{k + 1} }} } \right)I + t_{k + 1}^{{w_{k + 1} }} } + \left( {\prod\nolimits_{j = 1}^{k} {(t_{j} + v_{j} )^{{w_{j} }} - \prod\nolimits_{j = 1}^{k} {t_{j}^{{w_{j} }} } } } \right)I + \left( {(t_{k + 1} + v_{k + 1} )^{{w_{k + 1} }} - t_{k + 1}^{{w_{k + 1} }} } \right)\left( {\prod\nolimits_{j = 1}^{k} {(t_{j} + v_{j} )^{{w_{j} }} - \prod\nolimits_{j = 1}^{k} {t_{j}^{{w_{j} }} } } } \right)I}} \\ & = {l}_{{\prod\nolimits_{j = 1}^{k + 1} {t_{j}^{{w_{j} }} + \left( {\prod\nolimits_{j = 1}^{k + 1} {(t_{j} + v_{j} )^{{w_{j} }} } - \prod\nolimits_{j = 1}^{k + 1} {t_{j}^{{w_{j} }} } } \right)I} }} . \\ \end{aligned} $$

Therefore, according to the above results, we have Eq. (5) for any *n*. This completes the proof.□

Especially when *w*_*j*_ = 1/*n* for *j* = 1, 2, …, *n*, the NLNWGA operator is reduced to a NLN geometric average operator.

Then, the NLNWGA operator also shows the following properties:

1. Idempotency: Let $\bar{l}_{j}$ (*j* = 1, 2, …, *n*) be a collection of NLNs. Then there is $NLNWGA\left( {\bar{l}_{1} ,\bar{l}_{2} , \ldots ,\bar{l}_{n} } \right) = \bar{l}$ if $\bar{l}_{j}$ (*j* = 1, 2, …, *n*) is equal, i.e., $\bar{l}_{j}$ = $\bar{l}$ for *j* = 1, 2, …, *n*.
2. Monotonicity: Let $\bar{l}_{j}$ (*j* = 1, 2, …, *n*) be a collection of NLNs. Then there is $NLNWGA\left( {\bar{l}_{1} ,\bar{l}_{2} , \ldots ,\bar{l}_{n} } \right) \le NLNWGA\left( {\bar{l}_{1}^{*} ,\bar{l}_{2}^{*} , \ldots ,\bar{l}_{n}^{*} } \right)$ if $\bar{l}_{j} \le \bar{l}_{j}^{*}$ for *j* = 1, 2, …, *n*.
3. Boundedness: Let $\bar{l}_{j}$ (*j* = 1, 2, …, *n*) be a collection of NLNs and $\bar{l}_{{\rm min} } = \hbox{min} (\bar{l}_{1} ,\bar{l}_{2} , \ldots ,\bar{l}_{n} )$ and $\bar{l}_{{\rm max} } = \hbox{max} (\bar{l}_{1} ,\bar{l}_{2} , \ldots ,\bar{l}_{n} )$ for *j* = 1, 2, …, *n*, then there is $\bar{l}_{{\rm min} } \le NLNWGA\left( {\bar{l}_{1} ,\bar{l}_{2} , \ldots ,\bar{l}_{n} } \right) \le \bar{l}_{{\rm max} }$.
  Since the above properties are obvious, their proofs are omitted here.

## MAGDM method using the NLNWAA and NLNWGA operators

In this section, we present a handling method for MAGDM problems by using the NLNWAA and NLNWGA operators.

For a MAGDM problem with NLNs, let *U* = {*u*_1_, *u*_2_, …, *u*_*m*_} be a discrete set of alternatives, *G* = {*g*_1_, *g*_2_, …, *g*_*n*_} be a set of attributes, and *E* = {*e*_1_, *e*_2_, …, *e*_*p*_} be a set of DMs. If the *k*th (*k* = 1, 2,…, *p*) DM provides the evaluation of the alternative *u*_*i*_ (*i* = 1, 2, …, *m*) on the attribute *g*_*j*_ (*j* = 1, 2,…, *n*) under some linguistic term set, such as *L* = {*L*_0_: extremely poor, *L*_1_: very poor, *L*_2_: poor, *L*_3_: medium, *L*_4_: good, *L*_5_: very good, *L*_6_: extremely good}, the evaluation value with indeterminacy *I* can be represented by the form of a NLN $\bar{l}_{ij}^{k} = l_{{t_{ij}^{k} + v_{ij}^{k} I}}$ for $t_{ij}^{k} ,v_{ij}^{k} \in R$ (*k* = 1, 2,…, *p*; *j* = 1, 2,…, *n*; *i* = 1, 2,…, *m*). Therefore, we can obtain the *k*th NLN decision matrix *D* ^*k*^:

$$ D^{k} = \left[ {\begin{array}{*{20}c} {\bar{l}_{11}^{k} } &\quad {\bar{l}_{12}^{k} } &\quad  \cdots &\quad  {\bar{l}_{1n}^{k} } \\ {\bar{l}_{21}^{k} } & \quad {\bar{l}_{22}^{k} } & \quad \cdots & \quad {\bar{l}_{2n}^{k} } \\ \vdots & \quad \vdots & \quad \ddots & \quad \vdots \\ {\bar{l}_{m1}^{k} } & \quad {\bar{l}_{m2}^{k} } & \quad \cdots & \quad {\bar{l}_{mn}^{k} } \\ \end{array} } \right]. $$

If the weight vector of attributes is *W* = (*w*_1_, *w*_2_, …, *w*_*n*_) satisfying *w*_*j*_ ≥ 0 and $\sum\nolimits_{j = 1}^{n} {w_{j} = 1}$, and the weight vector of DMs is *Q* = (*q*_1_, *q*_2_,…, *q*_*p*_) satisfying *q*_*k*_ ≥ 0 and $\sum\nolimits_{k = 1}^{p} {q_{k} = 1}$. Then, the steps of the MADM problem are described as follows:

**Step 1** According to the decision matrix *D* ^*k*^ (*k* = 1, 2, …, *p*) provided by DMs, by the following formula:

8 $$ \bar{l}_{ij} = NLNWAA\left( {\bar{l}_{ij}^{1} ,\bar{l}_{ij}^{2} , \ldots ,\bar{l}_{ij}^{p} } \right) = l_{{\sum\nolimits_{k = 1}^{p} {q_{k} t_{ij}^{k} + I\sum\nolimits_{k = 1}^{p} {q_{k} v_{ij}^{k} } } }} , $$

we can get a collective NLN decision matrix:

$$ D = \left[ {\begin{array}{*{20}c} {\bar{l}_{11}^{{}} } &\quad {\bar{l}_{12}^{{}} } &\quad \cdots & \quad{\bar{l}_{1n}^{{}} } \\ {\bar{l}_{21}^{{}} } &\quad {\bar{l}_{22}^{{}} } &\quad \cdots & \quad{\bar{l}_{2n}^{{}} } \\ \vdots & \quad\vdots & \quad\ddots & \quad\vdots \\ {\bar{l}_{m1}^{{}} } &\quad {\bar{l}_{m2}^{{}} } & \quad\cdots & \quad{\bar{l}_{mn}^{{}} } \\ \end{array} } \right]. $$

**Step 2** The individual overall NLN $\bar{l}_{i}$ for *u*_*i*_ (*i* = 1, 2, …, *m*) is calculated by the following aggregation formula:

9 $$ \bar{l}_{i} = NLNWAA\left( {\bar{l}_{i1} ,\bar{l}_{i2} , \ldots ,\bar{l}_{in} } \right) = {l}_{{\sum\nolimits_{j = 1}^{n} {w_{j} t_{ij} + I\sum\nolimits_{j = 1}^{n} {w_{j} v_{ij} } } }} , $$

or

10 $$ \bar{l}_{i} = NLNWGA(\bar{l}_{i1} ,\bar{l}_{i2} , \ldots ,\bar{l}_{in} ) = {l}_{{\prod\nolimits_{j = 1}^{n} {t_{ij}^{{w_{j} }} + \left( {\prod\nolimits_{j = 1}^{n} {(t_{ij} + v_{ij} )^{{w_{j} }} } - \prod\nolimits_{j = 1}^{n} {t_{ij}^{{w_{j} }} } } \right)I} }} . $$

**Step 3** We introduce a de-neutrosophication process in the decision-making problem based on *I* ∈ [*inf I*, *sup I*] ⊂ [−1, 1]. A NLN $\bar{l}_{i}$ (*i* = 1, 2, …, *m*) can be transformed to an interval NLN, which is equivalent to $\bar{l}_{i} = l_{{t_{i} + v_{i} I}}$ ∈ $l_{{[t_{i} + v_{i} \inf I,t_{i} + v_{i} \sup I]}}$. Then, the expected value of *E*($\bar{l}_{i}$) (*i* = 1, 2, …, *m*) is calculated by applying Eq. (1).

**Step 4** The alternatives are ranked according to the values of *E*($\bar{l}_{i}$) (*i* = 1, 2, …, *m*) by the ranking method in Definition 3, and then the best one(s) can be selected according to the largest expected value of *E*($\bar{l}_{i}$).

**Step 5** End.

## Illustrative example

In this section, an illustrative example for a MAGDM problem with NLNs is provided to demonstrate the applications of the proposed decision-making method in realistic scenarios.

There is a decision-making problem of manufacturing alternatives in the flexible manufacturing system. Suppose a set of four alternatives for the flexible manufacturing system is *U* = {*u*_1_, *u*_2_, *u*_3_, *u*_4_}. Then, a decision is made according to the three attributes: (1) *g*_1_ is the improvement of manufacturing quality; (2) *g*_2_ is the market response; (3) *g*_3_ is the manufacturing cost. The four possible alternatives on the three attributes are to be evaluated by a group of three DMs corresponding to the linguistic term set *L* = {*L*_0_: extremely poor, *L*_1_: very poor, *L*_2_: poor, *L*_3_: medium, *L*_4_: good, *L*_5_: very good, *L*_6_: extremely good}, where DMs may contain the linguistic evaluation with indeterminacy *I* expressed by NLNs according to the linguistic term set. Assume that the weight vector of the three attributes is ***W*** = (0.2, 0.5, 0.3) and the weight vector of the three DMs is ***Q*** = (0.3, 0.36, 0.34).

Then, the three DMs are invited to make judgments and to give the linguistic evaluation with indeterminacy *I* expressed by NLNs according to the linguistic term set. Thus, the evaluation results of an alternative *u*_*i*_ (*i* = 1, 2, 3, 4) on an attribute *g*_*j*_ (*j* = 1, 2, 3) are given as the following three NLN decision matrices:

$$ D^{1} = \left[ {\begin{array}{*{20}c} {l_{5} } &\quad {l_{4 + I} } &\quad {l_{3 + I} } \\ {l_{4} } &\quad {l_{5} } &\quad {l_{4 + I} } \\ {l_{4 + I} } &\quad {l_{4 + I} } &\quad {l_{4} } \\ {l_{5} } &\quad {l_{4 + I} } &\quad {l_{4} } \\ \end{array} } \right],\quad D^{2} = \left[ {\begin{array}{*{20}c} {l_{4 + I} } &\quad {l_{5} } &\quad {l_{3} } \\ {l_{5} } &\quad {l_{4} } &\quad {l_{3 + I} } \\ {l_{5} } &\quad {l_{4 + I} } &\quad {l_{4} } \\ {l_{4 + I} } &\quad {l_{5} } & \quad{l_{5 + I} } \\ \end{array} } \right],\quad D^{3} = \left[ {\begin{array}{*{20}c} {l_{5 + I} } &\quad {l_{4} } &\quad {l_{3 + I} } \\ {l_{4 + I} } &\quad {l_{4} } &\quad {l_{3} } \\ {l_{5} } &\quad {l_{5} } &\quad {l_{4 + I} } \\ {l_{4} } &\quad {l_{4 + I} } &\quad {l_{4} } \\ \end{array} } \right]. $$

Whereas, we use the developed approach to rank the alternatives and to select the most desirable one(s), which can be described as the following steps:

**Step 1** According to the above three decision matrices of *D* ^*k*^ (*k* = 1, 2, 3), the collective NLN decision matrix is obtained by applying Eq. (8) as follows:

$$ D = \left[ {\begin{array}{*{20}c} {l_{4.64 + 0.7I} } &\quad {l_{4.36 + 0.3I} } &\quad {l_{3 + 0.64I} } \\ {l_{4.36 + 0.34I} } &\quad {l_{4.3} } &\quad {l_{3.3 + 0.66I} } \\ {l_{4.7 + 0.3I} } &\quad {l_{4.34 + 0.66I} } & \quad{l_{4 + 0.34I} } \\ {l_{4.3 + 0.36I} } & \quad{l_{4.36 + 0.64I} } & \quad{l_{4.36 + 0.36I} } \\ \end{array} } \right]. $$

**Step 2** By applying Eq. (9), we can obtain the individual overall NLNs of $\bar{l}_{i}$ for *u*_*i*_ (*i* = 1, 2, 3, 4):

$$ \bar{l}_{1} = l_{4.008+0.482I}, \;\bar{l}_{2} = l_{4.012+0.266I},\; \bar{l}_{3} = l_{4.31+0.492I},\;\; {\text{and}}\;\; \bar{l}_{4} = l_{4.348+0.5I}$$

**Step 3** For the de-neutrosophication in the decision-making problem, assume that the infimum of *I* is taken as *inf I* = 0 and the supremum of *I* is taken as *sup I* = 0.1 to consider the minimum and maximum values for indeterminacy *I*, which are determined by DMs’ preference or requirements in real situations. Thus by applying Eq. (1), we can obtain the expected values of *E*($\bar{l}_{i}$) (*i* = 1, 2, 3, 4):

$$E(  \bar{l}_{1} ) = 0.672,\; E(  \bar{l}_{2} ) = 0.6709,\; E(  \bar{l}_{3}) = 0.7224,\;\; {\text{and}}\;\; E(  \bar{l}_{4} ) = 0.7288.$$

**Step 4** Since *E*($\bar{l}_{4}$) > *E*($\bar{l}_{3}$) > *E*($\bar{l}_{1}$) > *E*($\bar{l}_{2}$), the ranking of four alternatives is *u*_4_ ≻ *u*_3_ ≻ *u*_1_ ≻ *u*_2_. Therefore, we can see that the alternative *u*_4_ is the best choice among all the alternatives.

Or we can also utilize the NLNWGA operator as the following computational steps:

**Step 1′** It is the same result as Step 1.

**Step 2′** By applying Eq. (9), we can obtain the individual overall NLNs of $\bar{l}_{i}$ for *u*_*i*_ (*i* = 1, 2, 3, 4):

$$\bar{l}_{1} = l_{3.9462+0.5004I},\; \bar{l}_{2} = l_{3.9828+0.2876I},\; \bar{l}_{3} = l_{4.3031+0.489I}, \;\;{\text{and}}\;\; \bar{l}_{4} = l_{4.3479+0.4976I}$$

**Step 3′** By applying Eq. (1) for *I* ∈ [0, 0.1], we can obtain the expected values of *E*($\bar{l}_{i}$) (*i* = 1, 2, 3, 4):

$$E( \bar{l}_{1} ) = 0.6619, \;E( \bar{l}_{2} ) = 0.6662, \;E(\bar{l}_{3}) = 0.7213, \;\;{\text{and}}\;\; E( \bar{l}_{4} ) = 0.7288.$$

**Step 4′** Since *E*($\bar{l}_{4}$) > *E*($\bar{l}_{3}$) > *E*($\bar{l}_{2}$) > *E*($\bar{l}_{1}$), the ranking of four alternatives is *u*_4_ ≻ *u*_3_ ≻ *u*_2_ ≻ *u*_1_. Therefore, we can see that the alternative *u*_4_ is the best choice among all the alternatives.

Similarly, if one considers different ranges of the indeterminate degree for *I* in NLNs, by Steps 3 and 4 or Steps 3′ and 4′, one can obtain different results, as shown in Tables 1 and 2.

**Table 1:** Decision results based on the NLNWAA operator by choosing different indeterminate ranges for *I* in NLNs

*I*	NLNWAA	Ranking
*I* ∈ [−0.7, 0]	*E*($\bar{l}_{1}$) = 0.6399, *E*($\bar{l}_{2}$) = 0.6532, *E*($\bar{l}_{3}$) = 0.6896, *E*($\bar{l}_{4}$) = 0.6955	*u*_4_ ≻ *u*_3_ ≻ *u*_2_ ≻ *u*_1_
*I* ∈ [−0.5, 0]	*E*($\bar{l}_{1}$) = 0.6479, *E*($\bar{l}_{2}$) = 0.6576, *E*($\bar{l}_{3}$) = 0.6978, *E*($\bar{l}_{4}$) = 0.7038	*u*_4_ ≻ *u*_3_ ≻ *u*_2_ ≻ *u*_1_
*I* ∈ [−0.3, 0]	*E*($\bar{l}_{1}$) = 0.6559, *E*($\bar{l}_{2}$) = 0.6620, *E*($\bar{l}_{3}$) = 0.7060, *E*($\bar{l}_{4}$) = 0.7122	*u*_4_ ≻ *u*_3_ ≻ *u*_2_ ≻ *u*_1_
*I* ∈ [−0.1, 0]	*E*($\bar{l}_{1}$) = 0.6640, *E*($\bar{l}_{2}$) = 0.6665, *E*($\bar{l}_{3}$) = 0.7142, *E*($\bar{l}_{4}$) = 0.7205	*u*_4_ ≻ *u*_3_ ≻ *u*_2_ ≻ *u*_1_
*I* = 0	*E*($\bar{l}_{1}$) = 0.6680, *E*($\bar{l}_{2}$) = 0.6687, *E*($\bar{l}_{3}$) = 0.7183, *E*($\bar{l}_{4}$) = 0.7247	*u*_4_ ≻ *u*_3_ ≻ *u*_2_ ≻ *u*_1_
*I* ∈ [0, 0.1]	*E*($\bar{l}_{1}$) = 0.6720, *E*($\bar{l}_{2}$) = 0.6709, *E*($\bar{l}_{3}$) = 0.7224, *E*($\bar{l}_{4}$) = 0.7288	*u*_4_ ≻ *u*_3_ ≻ *u*_1_ ≻ *u*_2_
*I* ∈ [0, 0.3]	*E*($\bar{l}_{1}$) = 0.6801, *E*($\bar{l}_{2}$) = 0.6753, *E*($\bar{l}_{3}$) = 0.7306, *E*($\bar{l}_{4}$) = 0.7372	*u*_4_ ≻ *u*_3_ ≻ *u*_1_ ≻ *u*_2_
*I* ∈ [0, 0.5]	*E*($\bar{l}_{1}$) = 0.6881, *E*($\bar{l}_{2}$) = 0.6797, *E*($\bar{l}_{3}$) = 0.7388, *E*($\bar{l}_{4}$) = 0.7455	*u*_4_ ≻ *u*_3_ ≻ *u*_1_ ≻ *u*_2_
*I* ∈ [0, 0.7]	*E*($\bar{l}_{1}$) = 0.6961, *E*($\bar{l}_{2}$) = 0.6842, *E*($\bar{l}_{3}$) = 0.7470, *E*($\bar{l}_{4}$) = 0.7538	*u*_4_ ≻ *u*_3_ ≻ *u*_1_ ≻ *u*_2_

**Table 2:** Decision results based on the NLNWGA operator by choosing different indeterminate ranges for *I* in NLNs

*I*	NLNWGA	Ranking
*I* ∈ [−0.7, 0]	*E*($\bar{l}_{1}$) = 0.6285, *E*($\bar{l}_{2}$) = 0.6470, *E*($\bar{l}_{3}$) = 0.6887, *E*($\bar{l}_{4}$) = 0.6956	*u*_4_ ≻ *u*_3_ ≻ *u*_2_ ≻ *u*_1_
*I* ∈ [−0.5, 0]	*E*($\bar{l}_{1}$) = 0.6369, *E*($\bar{l}_{2}$) = 0.6518, *E*($\bar{l}_{3}$) = 0.6968, *E*($\bar{l}_{4}$) = 0.7039	*u*_4_ ≻ *u*_3_ ≻ *u*_2_ ≻ *u*_1_
*I* ∈ [−0.3, 0]	*E*($\bar{l}_{1}$) = 0.6452, *E*($\bar{l}_{2}$) = 0.6566, *E*($\bar{l}_{3}$) = 0.7050, *E*($\bar{l}_{4}$) = 0.7122	*u*_4_ ≻ *u*_3_ ≻ *u*_2_ ≻ *u*_1_
*I* ∈ [−0.1, 0]	*E*($\bar{l}_{1}$) = 0.6535, *E*($\bar{l}_{2}$) = 0.6614, *E*($\bar{l}_{3}$) = 0.7131, *E*($\bar{l}_{4}$) = 0.7205	*u*_4_ ≻ *u*_3_ ≻ *u*_2_ ≻ *u*_1_
*I* = 0	*E*($\bar{l}_{1}$) = 0.6577, *E*($\bar{l}_{2}$) = 0.6638, *E*($\bar{l}_{3}$) = 0.7172, *E*($\bar{l}_{4}$) = 0.7247	*u*_4_ ≻ *u*_3_ ≻ *u*_2_ ≻ *u*_1_
*I* ∈ [0, 0.1]	*E*($\bar{l}_{1}$) = 0.6619, *E*($\bar{l}_{2}$) = 0.6662, *E*($\bar{l}_{3}$) = 0.7213, *E*($\bar{l}_{4}$) = 0.7288	*u*_4_ ≻ *u*_3_ ≻ *u*_2_ ≻ *u*_1_
*I* ∈ [0, 0.3]	*E*($\bar{l}_{1}$) = 0.6702, *E*($\bar{l}_{2}$) = 0.6710, *E*($\bar{l}_{3}$) = 0.7294, *E*($\bar{l}_{4}$) = 0.7371	*u*_4_ ≻ *u*_3_ ≻ *u*_2_ ≻ *u*_1_
*I* ∈ [0, 0.5]	*E*($\bar{l}_{1}$) = 0.6786, *E*($\bar{l}_{2}$) = 0.6758, *E*($\bar{l}_{3}$) = 0.7376, *E*($\bar{l}_{4}$) = 0.7454	*u*_4_ ≻ *u*_3_ ≻ *u*_1_ ≻ *u*_2_
*I* ∈ [0, 0.7]	*E*($\bar{l}_{1}$) = 0.6869, *E*($\bar{l}_{2}$) = 0.6806, *E*($\bar{l}_{3}$) = 0.7457, *E*($\bar{l}_{4}$) = 0.7537	*u*_4_ ≻ *u*_3_ ≻ *u*_1_ ≻ *u*_2_

For the decision results based on the NLNWAA operator in Table 1, we can see that the ranking orders of the four alternatives are *u*_4_ ≻ *u*_3_ ≻ *u*_2_ ≻ *u*_1_ from *I* ∈ [−0.7, 0] to *I* = 0 and *u*_4_ ≻ *u*_3_ ≻ *u*_1_ ≻ *u*_2_ from *I* ∈ [0, 0.1] to *I* ∈ [0, 0.7], and then the best alternative is *u*_4_. For the decision results based on the NLNWGA operator in Table 2, we can see that the ranking orders of the four alternatives are *u*_4_ ≻ *u*_3_ ≻ *u*_2_ ≻ *u*_1_ from *I* ∈ [−0.7, 0] to *I* ∈ [0, 0.3] and *u*_4_ ≻ *u*_3_ ≻ *u*_1_ ≻ *u*_2_ from *I* ∈ [0, 0.5] to *I* ∈ [0, 0.7], and then the best alternative is also *u*_4_. The illustrative example demonstrates that different ranges of indeterminate degrees for *I* in NLNs result in different ranking orders of alternatives. Then, the MAGDM method proposed in this paper can deal with the decision-making problems with NLN information (indeterminate linguistic information). If we do not consider the indeterminacy *I* in NLNs (i.e., *I* = 0), then this MAGDM method reduces to classical one with crisp linguistic values.

Furthermore, since the indeterminate linguistic part $l_{{v_{i} I}}$ in NLNs can affect the ranking order of alternatives in the MAGDM problem, the method proposed in this paper can provide more general and more flexible selecting way for DMs when the indeterminate degree for *I* in NLNs is assigned different ranges in de-neutrosophication process. Therefore, the DMs can select some ranges of indeterminate degrees for *I* in NLNs according to their preference or real requirements and have flexibility in real decision-making problems.

Obviously, the main advantage of the proposed method is able to express and handle indeterminate linguistic information in linguistic decision-making environments since the existing decision-making methods (Herrera et al. 1996; Herrera and Herrera-Viedma 2000; Xu 2006a, b; Ye 2015, 2016) cannot do it.

## Conclusion

This paper defined the operational laws of NLNs and the expected value of NLNs for ranking NLNs. Then, we proposed the NLNWAA and NLNWGA operators to aggregate NLN information and discussed their properties. Furthermore, a MAGDM method based on the NLNWAA and NLNWGA operators was established in NLN setting. Finally, an illustrative example demonstrated the application of the presented method. The proposed MAGDM method with NLNs is more suitable for real science and engineering applications because it easily express and handle the indeterminate linguistic information which exists commonly in real life. In the future research, we shall further develop other aggregation operators of NLNs, such as ordered weighted aggregation operators and prioritized weighted aggregation operators of NLNs, and apply them to assignment and resource allocation problems where the indeterminate information of the problems is specified uncertainly.

## References

[CR1] Herrera F, Herrera-Viedma E (2000). Linguistic decision analysis: steps for solving decision problems under linguistic information. Fuzzy Sets Syst.

[CR2] Herrera F, Herrera-Viedma E, Verdegay L (1996). A model of consensus in group decision making under linguistic assessments. Fuzzy Sets Syst.

[CR3] Kong L, Wu Y, Ye J (2015). Misfire fault diagnosis method of gasoline engines using the cosine similarity measure of neutrosophic numbers. Neutrosophic Sets Syst.

[CR4] Smarandache F (1998). Neutrosophy: neutrosophic probability, set, and logic.

[CR5] Smarandache F (2013). Introduction to neutrosophic measure, neutrosophic integral, and neutrosophic probability.

[CR6] Smarandache F (2014). Introduction to neutrosophic statistics.

[CR7] Smarandache F (2015). Symbolic neutrosophic theory.

[CR8] Xu ZS (2004). Uncertain linguistic aggregation operators based approach to multiple attribute group decision making under uncertain linguistic environment. Inf Sci.

[CR9] Xu ZS (2006). A note on linguistic hybrid arithmetic averaging operator in multiple attribute group decision making with linguistic information. Group Decis Negot.

[CR10] Xu ZS (2006). Goal programming models for multiple attribute decision making under linguistic setting. J Manag Sci China.

[CR11] Xu ZS (2006). Induced uncertain linguistic OWA operators applied to group decision making. Inf Fusion.

[CR12] Ye J (2015). Bidirectional projection method for multiple attribute group decision making with neutrosophic numbers. Neural Comput Appl.

[CR13] Ye J (2016). Multiple-attribute group decision-making method under a neutrosophic number environment. J Intell Syst.

[CR14] Zadeh LA (1975). The concept of a linguistic variable and its application to approximate reasoning Part I. Inf Sci.

